# Adult onset Still’s disease in a patient with scleroderma: case report

**DOI:** 10.1186/s41927-021-00212-4

**Published:** 2021-09-29

**Authors:** Jeffrey D. Brow, Daisy Zhu, Barbara E. Drevlow

**Affiliations:** 1 Department of Medicine, NorthShore University HealthSystem, 2650 Ridge Avenue, Evanston, IL 60201 USA; 2 Division of Rheumatology, Department of Medicine, NorthShore University HealthSystem, Evanston, IL USA

**Keywords:** Still's Disease, Adult-Onset, Scleroderma, Limited, Autoimmune Diseases, Case report

## Abstract

**Background:**

Scleroderma and adult onset Still’s disease (AOSD) are both uncommon autoimmune disorders. These two disorders have rarely been documented to occur simultaneously. In fact, after a thorough literature review, we discovered only one prior case report in a pregnant individual. Here, we describe the first documented case of scleroderma and AOSD in a postmenopausal patient.

**Case presentation:**

The patient is a 61-year-old Caucasian female with a past medical history significant for peptic ulcer disease, mitral valve prolapse, chronic idiopathic pancreatitis, and limited cutaneous scleroderma with sclerodactyly, Raynaud’s, and calcinosis. She was sent to the emergency room by her primary care physician due to one-week history of intermittent spiking fevers (Tmax 101**°**F), sore throat, myalgias, arthralgias, and non-pruritic bilateral lower extremity rash. Diagnostic evaluation in the hospital included complete blood count, comprehensive metabolic panel, respiratory viral panel, antinuclear antibody panel, bone marrow biopsy, and imaging with computerized tomography. Our patient fulfilled Yamaguchi Criteria for AOSD and all other possible etiologies were ruled out. She was treated with a steroid taper and methotrexate was initiated on post-discharge day number fourteen. Clinical and biochemical resolution was obtained at three months.

**Conclusions:**

In this report, we describe the first ever documented case of scleroderma and AOSD in a postmenopausal patient. The clinical presentation, diagnostic work up, and management discussed herein may serve as a framework for which rheumatologists and other physicians may draw upon in similar future encounters.

## Background

Systemic sclerosis, also known as scleroderma, is an uncommon autoimmune disease characterized by cutaneous fibrosis, vascular injury, and organ dysfunction. The etiology of this disorder is thought to be due to dysregulated repair of connective tissue in response to microcellular injury [1]. Two major subgroups exist (limited vs. diffuse) based upon the degree of cutaneous involvement. Patients in the limited subgroup are more prone to distal skin sclerosis, Raynaud’s phenomenon, and gastroesophageal reflux [2]. Those in the diffuse subgroup are more likely to experience proximal skin sclerosis and are at greater risk of developing dysfunction of one or more visceral organs (typically renal, cardiac, or pulmonary) [3].

Adult onset Still’s disease (AOSD) is another rare multisystem autoinflammatory disorder. It is characterized by high spiking fever (typically > 102.2°F), arthritis, splenomegaly, and rash. Joint pain is the most common symptom, and is most frequently observed in the wrists, knees, and ankles [4]. Maculopapular ‘salmon’ colored rash occurs in 60–80% of cases and commonly appears on the trunk or proximal limbs [5]. Laboratory analysis typically reveals a neutrophilic leukocytosis with elevated ESR, CRP, and ferritin levels greater than five times the limit of normal. The etiology of this disease is unknown, though infections and malignancies have been implicated as inciting factors [6, 7].

Scleroderma and AOSD are distinct disorders and have rarely been documented to occur simultaneously. The estimated annual incidence is 1.4–5.6 cases per 100,000 individuals for scleroderma and 0.16 cases per 100,000 individuals for AOSD [8, 9]. After a thorough literature review, we discovered only one prior case report of concurrent disease in a pregnant patient [10]. Here, we describe the first ever documented case of limited scleroderma and AOSD occurring together in a postmenopausal female.

## Case presentation

**Fig. 1:**
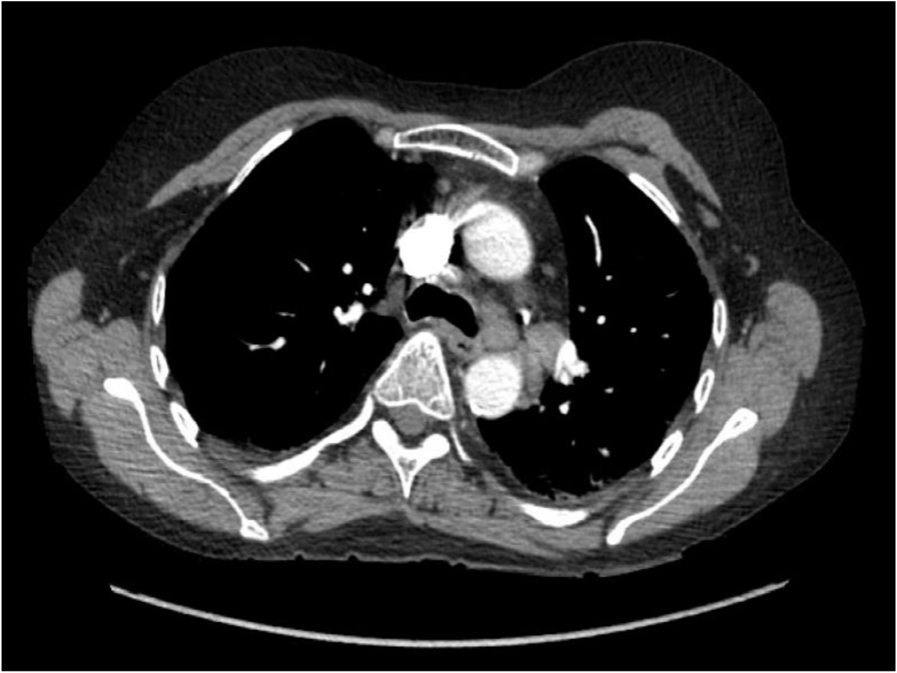
Mediastinal lymphadenopathy

**Fig. 2:**
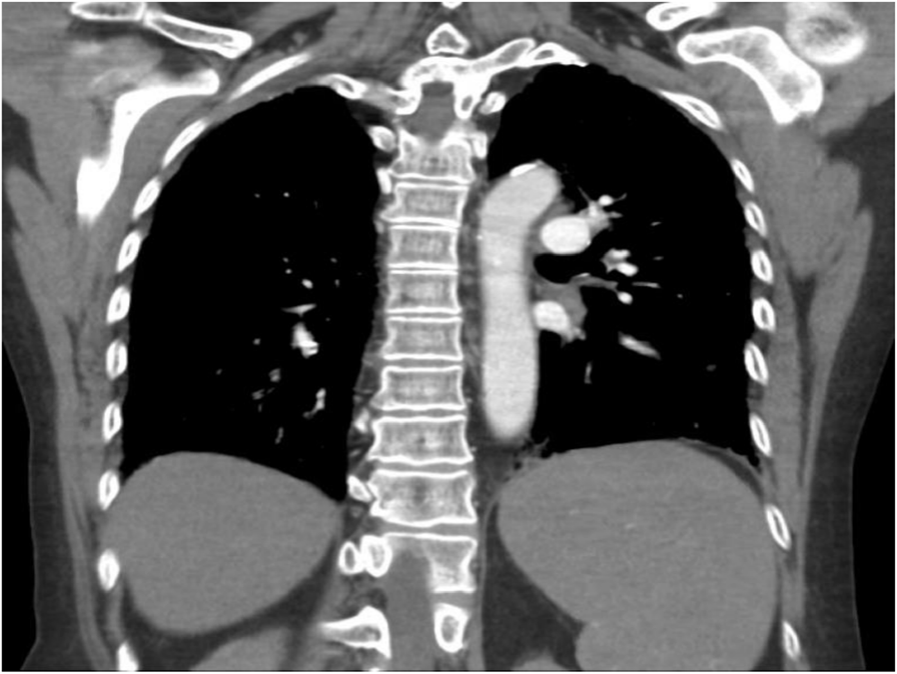
Splenomegaly

The patient presented to the emergency room with one-week history of sore throat, intermittent spiking fevers, myalgias, arthralgias, and non-pruritic bilateral lower extremity rash. Past medical history was significant for peptic ulcer disease, mitral valve prolapse, chronic idiopathic pancreatitis, and limited cutaneous scleroderma with manifestations of sclerodactyly, Raynaud’s, and calcinosis. Scleroderma had been diagnosed two decades prior. The patient had not been seen by a rheumatologist in four years, however she reported her skin and joint symptoms had been well controlled during this time. On physical exam, the patient was tachycardic (106), with palpable splenomegaly, and also had bilateral knee swelling with limited range of motion. Lab results were remarkable for leukocytosis (22,600) with leftward shift (88% PMNs), abnormal liver function tests—alkaline phosphatase (125), aspartate aminotransferase (50)—and elevated inflammatory markers—Ferritin (20,128), ESR (34), and CRP (206). There was no eosinophilia observed on the complete blood count. CT chest, abdomen and pelvis showed new mediastinal adenopathy and splenomegaly (Figs. 1 and 2). She was admitted to the hospital, and Infectious Disease, Rheumatology, and Hematology were consulted.

**Fig. 3:**
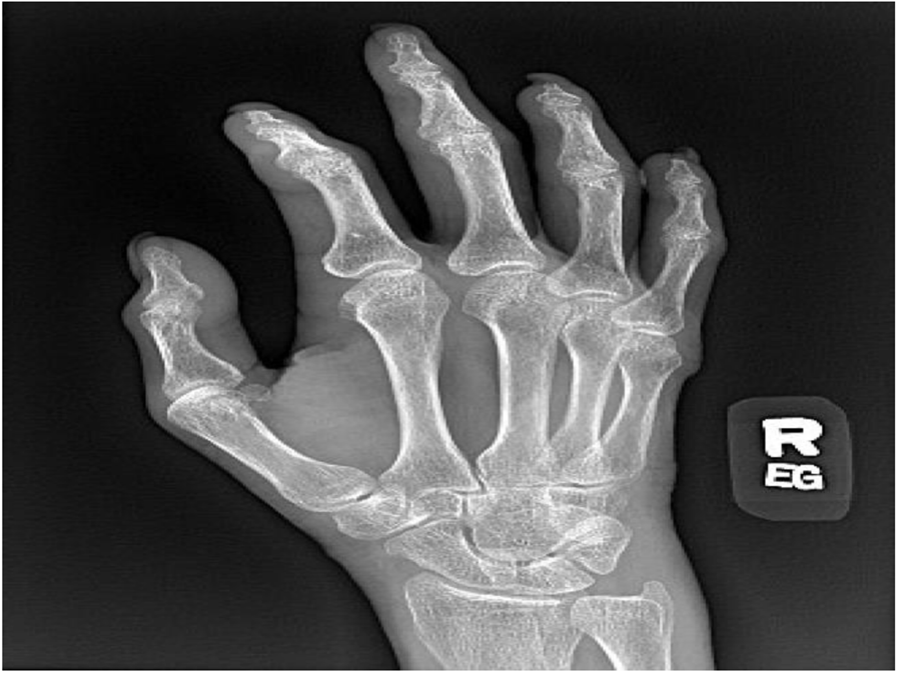
Juxta-articular soft tissue swelling with acro-osteolysis

While hospitalized, the patient's physical exam evolved from bilateral knee tenderness and swelling to bilateral ulnar wrist tenderness and swelling. X-rays of both wrists showed mild-to-moderate juxta-articular soft tissue swelling with acro-osteolysis (Fig. 3). Further laboratory testing was significant for negative rheumatoid factor, negative cyclic citrullinated peptide antibody, and elevated soluble CD 25 (1,933 pg/mL). The patient underwent a bone marrow biopsy to rule out hemophagocytic lymphohistiocytosis, which showed no evidence of such (Table 1). The patient was given four days of methylprednisone 20 mg as an inpatient, and discharged on a prednisone taper (fourteen days at 20 mg, ten days at 15 mg, fourteen days at 10 mg, 30 days at 5 mg). Methotrexate 12.5 mg weekly was initiated as steroid-sparing therapy on post-discharge day number fourteen. The patient’s symptoms and blood work were monitored closely with near complete resolution, both clinically and biochemically, at three months (Table 2). Liver function tests, specifically alkaline phosphatase and aspartate aminotransferase, had normalized prior to discharge from the hospital. Repeat Chest CT performed at six months demonstrated complete resolution of previous mediastinal adenopathy.

**Table 1:** Inpatient work up

Infectious	- Respiratory viral panel: negative - Blood cultures: no growth - Bone marrow cultures: no growth - Urinalysis: negative - Rapid strep test: negative - ASO titer: within normal limits - Hepatitis B: negative - Hepatitis C: negative - HIV: negative - CMV: IgM negative, IgG positive - EBV: IgM negative, IgG positive - Parvovirus B19: IgM negative, IgG positive
Rheumatologic	- ANA screen: negative - Rheumatoid factor: negative - Anti-CCP: negative - Anti-RNA polymerase III: negative - Anti-MPO/PR3: negative - Ferritin: > 20000 ng/mL (Ref: 11–306) - CRP: 206 mg/L (Ref: 0.0–4.9) - ESR: 34 mm/hr (Ref: 0.0–30) - Soluble CD 25: 1933 pg/mL (Ref: < 1033) - C3: 174.4 mg/dL (Ref: 81–157) - C4: 30.8 mg/dL (Ref: 12.9–39.2)
Hematologic	- Uric acid: 5.8 mg/dL (Ref: 2.4–5.7) - LDH: 730 U/L (Ref: 135–214) - Haptoglobin: 373 mg/dL (Ref: 16–200) - PT: 14.4 s (Ref: 9.4–11.8) - INR: 1.4 (Ref: 1.0) - PTT: 28 s (Ref: 22–30) - D-dimer: 3.65 mg/L (Ref: 0.0-.50) - Fibrinogen: 550 mg/dL (Ref: 173–417) - Bone marrow biopsy: hypercellular bone marrow with increased and left-shifted granulopoiesis with no evidence of hemophagocytosis

Results of the inpatient laboratory workup. *ASO* Antistreptolysin O. *CMV* Cytomegalovirus. *EBV* Epstein-Barr Virus. *ANA* Antinuclear Antibody. *CCP* Cyclic Citrullinated Peptide. *MPO/PR3* Myeloperoxidase/Proteinase 3. *CRP* C-Reactive Protein. *ESR* Erythrocyte Sedimentation Rate. *C3 & C4* Complement 3 & Complement 4. *LDH* Lactic Acid Dehydrogenase. *PT* Prothrombin Time. *INR* International Normalized Ratio. *PTT* Partial Thromboplastin Time. *Ref* Reference range

**Table 2:** Patient’s ferritin and c-reactive protein levels

	6/20/2018	6/25/2018	7/3/2018	7/10/2018	8/1/2018	9/26/2018	Reference Range
Ferritin (ng/mL)	20,128	5015	3593	3939	634	44	11–306
C-Reactive Protein (mg/L)	206.8	N/A	87.8	57.7	24.8	3.0	0.0–4.9

Ferritin & C-reactive protein levels trended from the patient’s initial presentation to the hospital through outpatient follow-up three months later

## Discussion and conclusion

Our patient had a history of limited scleroderma that was confirmed with positive ANA in a speckled and nucleolar pattern. She was taking pancrealipase for pancreatitis, diltiazem for Raynaud’s, celecoxib for arthralgia, and omeprazole due to her history of peptic ulcer disease. Regarding the patient’s chronic idiopathic pancreatitis, a thorough work up including abdominal imaging, tissue biopsy with histological analysis, and serum IgG levels had been completed at a prior date. There was no evidence to suggest a separate etiology, autoimmune or otherwise, to her recurrent pancreatitis.

The patient’s scleroderma had been controlled when she presented to the hospital with fever, sore throat, myalgias, arthralgias, and evanescent rash. Her symptoms began spontaneously with no preceding febrile illnesses, sick contacts, travel, or outdoor exposures. While AOSD primarily remains a diagnosis of exclusion, most studies utilize the Yamaguchi criteria due to its high sensitivity and specificity [11]. The criteria are fulfilled when a patient demonstrates at least five out of nine possible diagnostic features, with at least two being major criteria. Our patient’s symptoms (arthralgia, non-pruritic rash, and leukocytosis) fulfilled three major criteria per Yamaguchi. Her sore throat, lymphadenopathy, splenomegaly, and elevated liver function tests fulfilled four minor criteria. An important caveat to these criteria however, is that the presence of any infection, malignancy, or rheumatic disorder known to mimic AOSD precludes a diagnosis. Our patient’s workup was negative for infectious and malignant etiologies. Furthermore, she did not possess a diagnosis of any rheumatic disease known to mimic AOSD, such as rheumatoid arthritis, reactive arthritis, systemic lupus erythematosus, dermatomyositis, polymyositis, or vasculitis. In instances where AOSD is suspected to co-occur with one of these aforementioned disorders, it is important for the clinician to be aware that the Yamaguchi Criteria are not definitive in their ability to rule in or out AOSD. Therefore, physician intuition and special attention to clinical and biochemical clues will be paramount when making an accurate diagnosis.

Management of AOSD has been empirical, with data on treatment efficacy obtained from case reports and retrospective studies. Treatment varies according to disease severity [12]. Mild disease is managed with trial of NSAIDs to control fever and arthralgia. Management for moderate to severe disease is centered on glucocorticoids, with addition of anakinra or tocilizumab once the disease is controlled. Methotrexate can be used in patients with predominantly joint disease.

Development of systemic sclerosis is believed to occur in several distinct phases. The first of these phases is defined by immune mediated damage to vascular endothelial cells, particularly in arterioles [13, 14]. This damage leads to phase two, which is characterized by release of reactive oxygen species and monocytic infiltrate through gaps in endothelial cells into the perivascular space. Macrophages then release IL-8 and TGF-β that promote recruitment of fibroblasts as well as apoptosis of vascular smooth muscle cells [13, 15]. Th2 lymphocytes and CD20 positive B cells stimulate the release of IL-6 [1]. The cumulative effect of these processes leads to phase three, which consists of myofibroblast proliferation and eventual tissue hypoxia.

Less is known regarding the pathophysiology of AOSD. However, the process is thought to occur when a molecular signal, such as a pathogen or malignancy, activates a dysregulated inflammasome [4]. The inflammasome then releases IL-1 and IL-18. IL-18 leads to production of IFNγ, which in turn, increases macrophage activation. Simultaneously, IL-1 serves to recruit neutrophils and additional cytokines (such as IL-8, IL-6, and TNF-α) thereby creating a repetitive cycle of inflammation [4].

Although the clinical manifestations of limited cutaneous systemic sclerosis and AOSD are quite dissimilar, both conditions feature over activation of the innate immune system. In systemic sclerosis, macrophage dependent release of cytokines such as IL-6 and IL-8 contributes to a dysregulated inflammatory cascade that results in fibrosis. In AOSD, overproduction of many of these same cytokines leads to an inflammatory loop that produces symptoms of fever, rash, and joint pain.

In this report, we describe the first documented case of AOSD superimposed upon well-controlled scleroderma in a postmenopausal patient. Our patient presented with a known diagnosis of scleroderma and met seven of the Yamaguchi Criteria for AOSD (including three major criteria). She obtained prompt resolution of her symptoms with the use of glucocorticoids and methotrexate. It is unclear, however, why these two exceedingly rare conditions occurred together in the same individual, though it may suggest a heightened propensity for immune mediated inflammatory diseases in a subset of patients. Future studies may evaluate the potential for shared genetic foci between these, and other autoimmune conditions, with the hope of increasing diagnostic and therapeutic capabilities.

## Data Availability

All data generated and analyzed during this study are included in this published article.

## Abbreviations

AOSD: Adult onset Still’s disease
PMNs: Polymorphonuclear leukocytes
ESR: Erythrocyte sedimentation rate
CRP: C-reactive protein
CT: Computed tomography
ANA: Antinuclear antibody
IgG: Immunoglobulin G
NSAIDS: Nonsteroidal anti-inflammatory drugs
